# Incidence of Cigarette Smoking Relapse Among Individuals Who Switched to e-Cigarettes or Other Tobacco Products

**DOI:** 10.1001/jamanetworkopen.2021.28810

**Published:** 2021-10-19

**Authors:** John P. Pierce, Ruifeng Chen, Sheila Kealey, Eric C. Leas, Martha M. White, Matthew D. Stone, Sara B. McMenamin, Dennis R. Trinidad, David R. Strong, Tarik Benmarhnia, Karen Messer

**Affiliations:** 1 Herbert Wertheim School of Public Health and Human Longevity Science, University of California, San Diego, La Jolla; 2 Moores Cancer Center, University of California, San Diego, La Jolla; 3 Scripps Institution of Oceanography, University of California, San Diego, La Jolla

## Abstract

**Importance:**

Although e-cigarettes are not approved as a cessation device, many who smoke believe that e-cigarettes will help them quit cigarette smoking successfully.

**Objective:**

To assess whether people who recently quit smoking and who had switched to e-cigarettes or another tobacco product were less likely to relapse to cigarette smoking compared with those who remained tobacco free.

**Design, Setting, and Participants:**

This cohort study analyzed a nationally representative sample of US households that participated in 4 waves of the Population Assessment of Tobacco and Health Study (conducted 2013 through 2017), combining 2 independent cohorts each with 3 annual surveys. Eligible participants were individuals who smoked at baseline, had recently quit at the first follow-up, and completed the second follow-up survey.

**Exposures:**

Use of e-cigarettes or alternate tobacco products at follow-up 1 after recently quitting smoking.

**Main Outcomes and Measures:**

Weighted percentage of participants with over 12 months abstinence by follow-up 2.

**Results:**

Of a total of 13 604 participants who smoked cigarettes at baseline, 9.4% (95% CI, 8.7%-10.0%) recently had quit smoking (mean age, 41.9; 95% CI, 39.7-46.6 years; 641 [43.2%] women) Of these, 22.8% (95% CI, 19.7%-26.0%) had switched to e-cigarettes, with 17.6% (95% CI, 14.8%-20.5%) using them daily. A total of 37.1% (95% CI, 33.7%-40.4%) used a noncigarette tobacco product and 62.9% (95% CI, 59.6%-66.3%) were tobacco free. Rates of switching to e-cigarettes were highest for those who were in the top tertile of tobacco dependence (31.3%; 95% CI, 25.0%-37.7%), were non-Hispanic White (26.4%; 95% CI, 22.3%-30.4%), and had higher incomes (annual income ≥$35 000, 27.5%; 95% CI, 22.5%-32.4% vs <$35 000, 19.3%; 95% CI, 16.3%-22.3%). At follow-up 2, unadjusted relapse rates were similar among those who switched to different tobacco products (for any tobacco product: successfully quit, 41.5%; 95% CI, 36.2%-46.9%; relapsed with significant requit, 17.0%; 95% CI, 12.4%-21.6%; currently smoking, 36.2%; 95% CI, 30.9%-41.4%). Controlled for potential confounders, switching to any tobacco product was associated with higher relapse rate than being tobacco free (adjusted risk difference, 8.5%; 95% CI, 0.3%-16.6%). Estimates for those who switched to e-cigarettes, whether daily or not, were not significant. While individuals who switched from cigarettes to e-cigarettes were more likely to relapse, they appeared more likely to requit and be abstinent for 3 months at follow-up 2 (17.0%; 95% CI, 12.4%-21.6% vs 10.4%; 95% CI, 8.0%-12.9%).

**Conclusions and Relevance:**

This large US nationally representative study does not support the hypothesis that switching to e-cigarettes will prevent relapse to cigarette smoking.

## Key Points

**Question:**

Is switching to e-cigarettes associated with prevention of relapse to cigarette smoking?

**Findings:**

In this cohort study of a representative sample of US adults, 9.4% of respondents who smoked cigarettes became recent former smokers 1 year later. Switching to any tobacco product including e-cigarettes was associated with an 8.5% increase in relapse to smoking over the next year; this increase in relapse was similar to that seen in individuals who switched to other tobacco products.

**Meaning:**

Switching to e-cigarettes is not associated with relapse prevention for cigarette smoking in US adults.

## Teaser

This cohort study examines the relapse rates for adults who switched from smoking cigarettes to e-cigarettes or other tobacco products vs those who remained tobacco free.

## Introduction

Switching to less harmful sources of nicotine has long been advocated as a way to reduce the health consequences of cigarette smoking.^1^ e-Cigarettes provide a noncombustible source of nicotine, which allows for the inhalation of nicotine without many of the known carcinogens in cigarette smoke.^2^ Those who can switch to e-cigarettes without relapsing to cigarette smoking will reduce the health consequences of their nicotine addiction.^3^ There is evidence that some who smoke cigarettes can use e-cigarettes to help them quit cigarettes and not relapse, at least in the short term,^4,5^ with daily e-cigarette users potentially having lower relapse rates than those who did not switch to e-cigarettes.^6^

In 2016, 24.7% of individuals who smoke reported using e-cigarettes to help them quit cigarettes.^7^ Using e-cigarettes as part of a quit attempt is more common among those who are younger, less dependent on nicotine, more educated, have higher income, are from non-Hispanic White race and ethnicity, and those who believe e-cigarettes are less harmful than cigarettes.^8,9^ A number of these variables are also related to the probability that a quit attempt will lead to successful cessation,^10^ making them potential confounders that need to be controlled in any study of the effectiveness of e-cigarettes.

The potential for harm reduction with e-cigarettes requires that those attempting to quit successfully switch completely away from cigarettes and not become dual-product users.^2^ For some former smokers who had quit all tobacco, there is evidence that e-cigarettes might be an intermediate step in their relapse to cigarette smoking.^11,12^ No population studies to date have reported on the long-term success rates among recent former smokers who had switched from cigarettes to e-cigarettes or to other alternate tobacco products.

In this study, we use 4 annual waves of the US Population Assessment of Tobacco and Health (PATH) study^13^ to identify 2 separate cohorts of adults who had recently quit smoking cigarettes, with each cohort completing 3 sequential annual surveys. Our research design follows the best practice guidelines for studying e-cigarettes and cessation in observational studies.^2^ We measured potential confounders^8^ when respondents still smoked cigarettes, but our study population comprised recent former smokers at the next annual survey (follow-up 1, when we measured the alternate products to which they had switched). We used a third annual survey (follow-up 2) to assess whether individuals had relapsed to cigarette smoking. We tested whether (1) those who switched to another tobacco product or specifically e-cigarettes at follow-up 1 were less likely to relapse to cigarette smoking by follow-up 2 than those who were tobacco free and (2) switching to daily use of any tobacco product or specifically e-cigarettes at follow-up 1 was associated with lower relapse than those who were tobacco free.

## Methods

### Data Sources and Study Sample

The PATH study is a US nationally representative, longitudinal cohort study that recruited and surveyed a stratified sample of the noninstitutionalized, civilian population from September 2013 through December 2014 (wave 1) and conducted annual in-person follow-up surveys in the respondent’s place of residence. The PATH study oversampled African American individuals, tobacco users, and young people. Response rates were 54% for the initial household screener and 74.0% for the wave 1 adult survey, with follow-up conditional response rates of 83.1%, 78.4%, and 73.5% at waves 2 through 4, respectively. The Westat institutional review board approved the study, and respondents provided written informed consent. In this study we used deidentified data from publicly available restricted use files (National Addiction & HIV Data Archive Program)^14^ and followed the Strengthening the Reporting of Observational Studies in Epidemiology (STROBE) reporting guideline.

**Figure 1.**
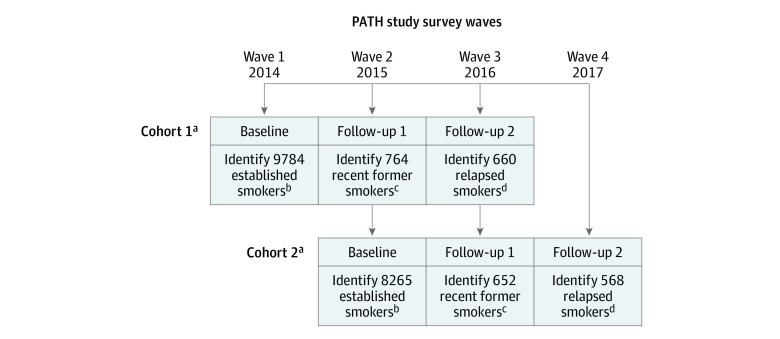
Study Design Using 2 PATH Study Cohorts Abbreviation: PATH, Population Assessment of Tobacco and Health. ^a^Cohorts were independent and each participant responded to 3 survey waves (baseline, follow-up 1, follow-up 2). ^b^Established smokers included survey respondents who had smoked 100 cigarettes in their lifetime, answered that they currently smoke cigarettes, and had smoked fairly regularly for 3 years. Those who participated in follow-up 1 and follow-up 2 surveys were included in all analyses. ^c^Recent former smokers included survey respondents who were not smoking cigarettes at follow-up 1 but who were established smokers at baseline. Those who participated in follow-up 2 survey were included in all analyses. ^d^Relapsed smokers included recent former smokers at follow-up 1 who, at follow-up 2, reported either that they were a current cigarette smoker or they responded positively to the question, “In the past 12 months, have you smoked a cigarette even 1 or 2 puffs?”

This study considered adults who smoked at baseline who identified as recent former smokers on the next annual survey (follow-up 1) and completed a third survey the following year (follow-up 2). Switching to a noncigarette tobacco product such as e-cigarettes or any other tobacco product was assessed at follow-up 1. To increase analytic power, we study 2 cohorts of recent former smokers: the first cohort spans waves 1 through 3 (2014 to 2016) and the second cohort spans waves 2 through 4 (2015 to 2017) (Figure 1). In this design, no member of the first cohort could be in the second cohort, and the combined cohort comprised 1228 recent former smokers.

### Measures

#### Cigarette Smoking Status

At each survey, adults who had smoked 100 or more cigarettes in their lifetime were asked if they “currently smoke every day, some days, or not at all,” and current smokers were asked, “About how long did you smoke fairly regularly?” Following Dai and Leventhal,^12^ we limited our population to adults who are established smokers (ie, 3 or more years of fairly regular smoking). From these established smokers, recent former smokers at follow-up 1 were those who answered “not at all” to the current smoking question or answered did not smoke in the past 12 months. Following previous work,^15,16^ we defined successfully quit at follow-up 2 as those who were completely abstinent for 12 months or more from the question, “In the past 12 months, have you smoked a cigarette even 1 or 2 puffs?” Individuals who relapsed were categorized as either current smokers or individuals who requit. We categorized the duration of the requit from the question, “About how long has it been since you last smoked a cigarette?”

#### Use of Noncigarette Tobacco Products at Follow-up 1

Our exposure of interest was current use of tobacco products by recent former smokers at follow-up 1. While there is disagreement on whether e-cigarettes should be labeled as tobacco products, as the US Food and Drug Administration does,^17^ for the purposes of this study we categorized them as such. On each survey, the PATH study queries current use (ie, responses of every day, some days, or not at all) for each of the following products: e-cigarettes, other e-products, cigars, cigarillo, filtered cigars, pipes, hookah, snus, and smokeless products.

#### Study Covariates

The PATH study questionnaire includes standard questions on sociodemographics including asking participants to choose all that apply from 4 categories of Latinx or Spanish ethnic origin and 14 categories of race. As it is not the goal of this article to provide estimates for each race or ethnicity, and because the distribution of 378 participants across 4 ethnic categories and 14 categories of race resulted in small sample sizes, we used the racial category with the largest sample, non-Hispanic White. In previous reports of e-cigarettes and cessation in the PATH study^8,18^ we identified the following additional potential confounders: cigarette consumption at baseline, duration of cigarette abstinence, smoke-free home, perceived harmfulness of cigarettes, relative perceived harmfulness of e-cigarettes, exposure to others who smoke, cigarette pack-years, age began regular smoking, existence of smoking-related disease, internal mental health symptoms, external mental health symptoms, and insurance status, as well as a 16-item tobacco dependence index previously shown to be reliable and valid in projecting tobacco use behavior (eAppendix in the Supplement).^19^ All variables were measured on the baseline survey except for smoke-free home and duration of cigarette abstinence, which were assessed at follow-up 1.

### Statistical Analyses

All analyses were conducted in R version 3.5.3 (R Project for Statistical Computing). Estimates were weighted using survey longitudinal weights, which accounted for study drop-outs (ie, wave 1 through 3 weights for cohort 1 and wave 2 through 4 weights for cohort 2).^14^ Variance estimates for confidence intervals and *P* values were calculated using replicate weights constructed with a balanced repeated replications procedure with Fay adjustment (ρ = 0.3). Sample characteristics were estimated using weighted proportions with 95% confidence limits (eTable in the Supplement).

We used multivariable logistic regression to estimate the marginal risk differences adjusting on all identified covariates. Missing data in those covariates were imputed assuming missing-at-random patterns and using default multiple imputation models, with 20 imputed data sets generated. For each imputed data set, we conducted logistic regression with longitudinal survey weights and replicated weights generated with the R package zelig.^14^ The marginal risk difference was derived based on logistic regression fitted values. Finally, we used Rubin’s Rule^20^ to derive the estimated marginal risk difference and its standard error based on the 20 imputed data sets. All statistical tests were 2-sided and we used 95% confidence intervals to assess statistical significance at *P* < .05.

## Results

Overall, 13 604 out of 18 049 adults considered current established smokers completed both follow-up 1 and follow-up 2. More established smokers were identified in cohort 1 but there was no difference in terms of drop-out rates between cohorts (Figure 1). The mean age of recent former smokers was 41.9 years (95% CI, 40.8-43.0 years) and 641 (43.2%) were women; 62.9% (95% CI, 59.6%-66.3%) were tobacco free at follow-up 1 and 22.8% (95% CI, 19.7%-26.0%) had switched to e-cigarettes with 17.6% (95% CI, 14.8%-20.5%) being daily users (Table 1). These proportions were similar between cohorts. Some 10.5% (95% CI, 8.4%-12.6%) had switched to various types of cigars with one third reporting daily use. A total of 9.7% (95% CI, 7.3%-12.1%) had switched to one of a variety of other tobacco products with half switching to daily use.

**Table 1.** Contribution of Each Cohort to Study Samples

Characteristic	Cohort 1^a^		Cohort 2^b^		Combined sample	
	No.	Weighted % (95% CI)	No.	Weighted % (95% CI)	No.	Weighted % (95% CI)
Study sample						
Established smokers at baseline^c^	7089	NA	6515	NA	13 604	NA
RFS at follow-up 1 who completed follow-up 2 ^d^	660	9.6 (8.8-10.4)	568	9.1 (8.3-10.0)	1228	9.4 (8.7-10.0)
Exposure of RFS assessed at follow-up 1						
Any tobacco product or e-cigarette use	250	36.8 (32.5-41.1)	209	37.3 (32.4-42.2)	459	37.1 (33.7-40.4)
Any e-cigarette use	160	23.4 (19.1-27.7)	126	22.2 (18.3-26.2)	286	22.8 (19.7-26.0)
Daily tobacco product or e-cigarette use	169	24.7 (20.8-28.6)	142	26.5 (21.8-31.1)	311	25.6 (22.4-28.8)
Daily e-cigarette use	121	18.1 (14.3-21.9)	95	17.2 (13.6-20.8)	216	17.6 (14.8-20.5)
Any cigar use^e^	63	8.8 (6.2-11.4)	71	12.2 (9.4-15.1)	134	10.5 (8.4-12.6)
Any combustible tobacco product use^f^	83	11.6 (8.7-14.6)	79	13.8 (10.7-16.8)	162	12.7 (10.5-14.9)
No tobacco use	410	63.2 (58.9-67.5)	359	62.7 (57.8-67.6)	769	62.9 (59.6-66.3)

Abbreviations: NA, not applicable; PATH, Population Assessment of Tobacco and Health; RFS, recent former smoker.

^a^ Cohort 1 surveyed PATH waves 1 through 3, conducted 2014 to 2016 (Figure 1).

^b^ Cohort 2 surveyed PATH waves 2 through 4, 2015 to 2017 (Figure 1).

^c^ Respondents who at baseline reported to have smoked 100 cigarettes in their lifetime, currently smoked cigarettes, and had smoked fairly regularly for 3 years.

^d^ RFSs who were not smoking cigarettes at follow-up 1 but were established smokers at baseline and completed follow-up 2.

^e^ Any cigar use included any use of cigar, cigarillo, or filtered cigar.

^f^ Any combusted tobacco product use included any use of cigar, cigarillo, filtered cigar, pipe, or hookah.

### Characteristics of Recent Former Smokers by Tobacco Product Use at Follow-up 1

We estimated demographic and tobacco use characteristics among those recent former smokers who switched to another tobacco product (including e-cigarettes) compared with those who were tobacco-free and assess differences using non-overlapping confidence intervals (Table 2). Among respondents aged 18 to 34 years, 53.3% (95% CI, 47.3%-59.4%) were tobacco free at follow-up 1, which was lower than either of the 2 older age groups (eg, ages 35 to 50 years, 66.7%; 95% CI, 61.3%-72.2%); among respondents aged 18 to 34 years, 25.8% (95% CI, 20.9%-30.6%) had switched to e-cigarettes, which was not different than older age groups. Thus, the age difference in any noncigarette tobacco use (age 18 to 34 years, 46.7%; 95% CI, 40.6%-52.7% vs age 35 to 50 years, 33.3%; 95% CI, 27.8%-38.7%) came from higher use of other tobacco products.

**Table 2.** Characteristics of Recent Former Cigarette Smokers by Use of Noncigarette Tobacco Products at Follow-up 1^a^^,^^b^

Variable	Sample size	Weighted % (95% CI)		
		No tobacco product use (n = 769)	Any noncigarette tobacco use (n = 459)	Any e-cigarette use^c^ (n = 286)
Age, y				
18-34	546	53.3 (47.3-59.4)	46.7 (40.6-52.7)	25.8 (20.9-30.6)
35-50	351	66.7 (61.3-72.2)	33.3 (27.8-38.7)	23.1 (17.9-28.4)
≥50	331	72.4 (67.6-77.1)	27.7 (22.9-32.4)	18.5 (13.5-23.6)
Sex				
Men	641	56.3 (51.7-60.9)	43.7 (39.1-48.3)	25.1 (21.1-29.0)
Women	587	71.6 (67.5-75.8)	28.4 (24.2-32.5)	19.9 (15.7-24.1)
Education				
<High school	254	66.6 (60.5-72.8)	33.4 (27.2-39.5)	19.1 (13.9-24.3)
High school graduate	258	62.4 (54.9-69.8)	37.6 (30.2-45.1)	20.4 (14.8-26.0)
≥Some college	707	61.8 (57.4-66.2)	38.2 (33.8-42.6)	25.0 (20.9-29.1)
Race and non-Hispanic ethnicity				
White	809	59.4 (54.9-63.9)	40.6 (36.1-45.1)	26.4 (22.3-30.4)
Others^d^	378	69.9 (65.3-74.5)	30.1 (25.5-34.7)	15.1 (11.0-19.2)
Income (US), $				
<35 000	625	65.7 (61.8-69.6)	34.3 (30.4-38.2)	19.3 (16.3-22.3)
≥35 000	530	58.9 (53.7-64.1)	41.1 (35.9-46.3)	27.5 (22.5-32.4)
Tobacco dependence^e^				
0-33.3	441	71.3 (66.3-76.3)	28.7 (23.7-33.7)	14.9 (10.9-18.9)
33.4-66.7	490	61.0 (55.3-66.6)	39.0 (33.4-44.7)	25.4 (20.3-30.5)
66.8-100	295	52.6 (46.0-59.2)	47.4 (40.8-54.0)	31.3 (25.0-37.7)
Cigarette abstinence, d				
<90	442	60.8 (55.5-66.0)	39.3 (34.0-44.5)	24.3 (19.4-29.1)
≥90	775	64.4 (60.4-68.4)	35.6 (31.6-39.6)	22.1 (18.3-25.8)
Relative harm				
Less harmful	612	54.4 (50.2-58.7)	45.6 (41.3-49.8)	32.5 (28.1-36.9)
About the same	470	67.9 (63.1-72.7)	32.1 (27.3-36.9)	15.5 (11.6-19.3)
More harmful	84	76.7 (65.5-87.8)	23.3 (12.2-34.5)	8.5 (2.4-14.6)

Abbreviation: PATH, Population Assessment of Tobacco and Health.

^a^ Recent former smokers were survey respondents who were not smoking cigarettes at follow-up 1 but who were established smokers at baseline.

^b^ Noncigarette tobacco use included any use of e-cigarette, other e-products, cigar, cigarillo, filtered cigar, pipe, hookah, snus, or smokeless tobacco.

^c^ e-Cigarette included in the any non-cigarette tobacco use subgroup.

^d^ The PATH study questionnaire included standard questions on sociodemographics including asking participants to choose all that apply from 4 categories of Latinx or Spanish origin and 14 categories of race.

^e^ Tobacco Dependence Index tertiles based on Strong et al.^16^

Women were more likely to be tobacco free at follow-up 1 than men (71.6%; 95% CI, 67.5%-75.8% vs 56.3%; 95% CI, 51.7%-60.9%). There was no sex difference in use of e-cigarettes, indicating that the higher rates of men (43.7%; 95% CI, 39.1%-48.3% vs 28.4%; 24.2%-32.5%) using alternate tobacco products came from the use of products other than e-cigarettes. There were no differences by educational status in the use of products at follow-up 1. Non-Hispanic White respondents (59.4%; 95% CI, 54.9%-63.9%) were less likely to be tobacco-free at follow-up 1 than other race and ethnicity groups (69.9%; 95% CI, 65.3%-74.5%), and a difference partly owing to their higher use of e-cigarettes (White, 26.4%; 95% CI, 22.3%-30.4% vs all other Non-Hispanic populations, 15.1%; 95% CI, 11.0%-19.2%) with no additional difference for any alternate tobacco product. There was no difference by income level in the proportion who were tobacco free at follow-up 1; however, those with higher incomes (ie, greater than $35 000) had a higher prevalence of e-cigarette use compared with those with lower incomes (27.5%; 95% CI, 22.5%-32.4% vs 19.3%; 95% CI, 16.3%-22.3%).

For tobacco dependence (measured at baseline, when respondents were current established smokers), each higher tertile of dependence was associated with a lower prevalence of being tobacco free at follow-up 1 (lowest tertile, 71.3%; 95% CI, 66.3%-76.3% vs highest tertile, 52.6%; 95% CI, 46.0%-59.2%). Those in the lowest tertile of dependence were less likely to use an e-cigarette than those in the highest tertile (14.9%; 95% CI, 10.9%-18.9% vs 31.3%; 95% CI, 25.0%-37.7%). The upper 2 tertiles also had a higher prevalence of use of other tobacco products resulting in significantly higher overall prevalence of any tobacco use (lowest, 28.7%; 95% CI, 23.7%-33.7% vs highest, 47.4%; 95% CI, 40.8%-54.0%). There was no difference in use of tobacco products based on the length of time since the respondent had quit smoking at follow-up 1. Viewing e-cigarettes as less harmful (compared with perceiving them as having about the same level of harm as cigarettes) was associated with a lower prevalence of being tobacco free at follow-up 1 (less harm, 54.4%; 95% CI, 50.2%-58.7% vs about the same, 67.9%; 95% CI, 63.1%-72.7%). This difference came from a higher proportion of respondents with this viewpoint who switched to e-cigarettes (less harm, 32.5%; 95% CI, 28.1%-36.9% vs about the same, 15.5%; 95% CI, 11.6%-19.3%).

### Unadjusted Cigarette Smoking Status at Follow-up 2 by Product Used at Follow-up 1

Mean time between follow-up 1 and follow-up 2 was 12.1 months (95% CI, 10.5-16.6 months). In Table 3, we report smoking status at follow-up 2 for 7 categories (not mutually exclusive) of products used at follow-up 1. A higher proportion of those who were tobacco free at follow-up 1 (50.5%; 95% CI, 46.4%-54.6%) had successfully quit at follow-up 2 than those who used any tobacco product (41.5%; 95% CI, 36.2%-46.9%). There was no difference between any subgroup of tobacco or e-cigarette users who had successfully quit (range, 40.7%-42.8%). For respondents who were currently smoking at follow-up 2, there was no difference between any product use category at follow-up 1, including no tobacco use. While respondents who switched to e-cigarettes were more likely to relapse by follow-up 2, they were also more likely to requit for at least 3 months than those who did not use tobacco (17.0%; 95% CI, 12.4%-21.6% vs 10.4%; 95% CI, 8.0%-12.9%).

**Table 3.** Unadjusted Abstinence Status at Follow-up 2 Among Recent Former Cigarette Smokers, by Use of Noncigarette Tobacco Products Assessed at Follow-up 1^a^

Exposure as RFS assessed at follow-up 1	Sample size	Cigarette smoking status at follow-up 2, weighted % (95% CI)			
		Successfully quit (≥12 mo)^b^	Relapsed		
			Significant requit (3-12 mo)	Requit (0-3 mo)	Current smoker
Any tobacco product or e-cigarette use^c^	459	41.5 (36.2-46.9)	17.0 (12.4-21.6)	5.3 (3.2-7.4)	36.2 (30.9-41.4)
Any e-cigarette use	286	41.6 (34.7-48.5)	17.4 (11.9-22.9)	4.7 (2.1-7.2)	36.3 (30.6-42.0)
Daily tobacco product or e-cigarette use	311	41.3 (35.3-47.4)	19.3 (13.6-24.9)	5.1 (2.5-7.8)	34.3 (29.0-39.6)
Daily e-cigarette use	216	42.8 (35.7-49.8)	19.1 (12.8-25.3)	4.7 (1.7-7.7)	33.4 (27.4-39.5)
Any cigar use^d^	134	42.1 (32.2-52.0)	11.9 (5.2-18.6)	7.3 (2.8-11.8)	38.7 (28.3-49.2)
Any combusted tobacco product use^e^	162	40.7 (32.1-49.3)	14.7 (7.3-22.1)	7.0 (3.1-10.9)	37.6 (28.9-46.3)
No tobacco use	769	50.5 (46.4-54.6)	10.4 (8.0-12.9)	5.1 (3.6-6.5)	34.0 (30.2-37.9)

Abbreviation: RFS, recent former smoker.

^a^ Recent former smokers included survey respondents who were not smoking cigarettes at follow-up 1 but who were established smokers at baseline.

^b^ Successfully quitting was defined as complete abstinence for 12 months or more based on survey responses to the question, “In the past 12 months, have you smoked a cigarette even 1 or 2 puffs?”

^c^ Any tobacco product or e-cigarette use included any use of e-cigarette, other e-products, cigar, cigarillo, filtered cigar, pipe, hookah, snus, or smokeless tobacco.

^d^ Any cigar use included any use of cigar, cigarillo, or filtered cigar.

^e^ Any combusted tobacco product use included any use of cigar, cigarillo, filtered cigar, pipe, or hookah.

### Adjusted Risk Difference in Relapse to Smoking by Product Used at Follow-up 1

**Figure 2.**
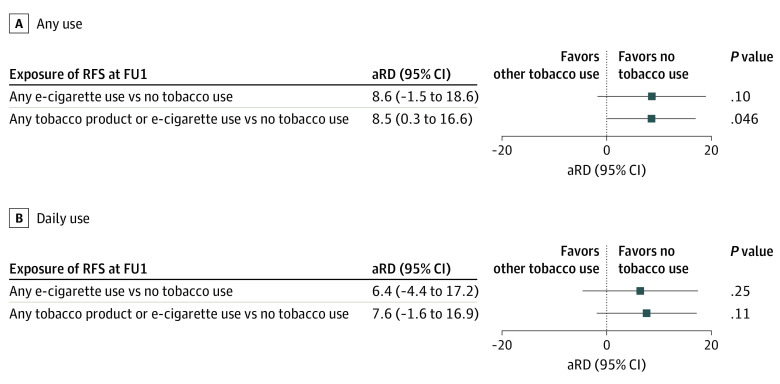
Adjusted Risk Difference of Relapse by Follow-up 2 for Recent Former Smokers by Product Used at Follow-up 1 Abbreviations: aRD, adjusted risk difference; RFS, recent former smokers. Analyses were adjusted for the following covariates: age, sex, education, race, ethnicity, income, nicotine dependence, cigarette consumption at baseline, duration of cigarette abstinence, smoke-free home, perceived harmfulness of cigarettes, relative perceived harmfulness of e-cigarettes, exposure to others who smoke, pack-years, age began regular smoking before 18, existence of smoking-related disease, internal mental health symptoms, external mental health symptoms, and insurance status. Missing data were imputed; 20 imputed sets were generated, and statistical inference was based on Rubin’s Rules.Any tobacco product included any use of other e-products, cigar, cigarillo, filtered cigar, pipe, hookah, snus, or smokeless tobacco.

Estimates from adjusted logistic regressions indicated that the relapse rate was 8.5% higher among those who switched to use any tobacco product compared with those who were tobacco-free at follow-up 1 (49.3% vs 57.8%; aRD, 8.5%; 95% CI, 0.3% to 16.6%). Results for those who switched to any e-cigarette use compared with those who were tobacco-free were not statistically significant (50.0% to 58.5%; aRD, 8.6%; 95% CI, −1.5% to 18.6%) (Figure 2). Considering those who were daily users of any product at follow-up 1, the relapse rate was not significantly different compared with those who were tobacco free (60.4% vs 52.8%; aRD, 7.6%; 95% CI, −1.6% to 16.9%). The risk difference was similar for respondents who were daily e-cigarette users, although again the confidence interval crossed zero (60.8% vs 54.4%; aRD, 6.4%; 95% CI, −4.4% to 17.2%).

## Discussion

In this study using a nationally representative cohort, we did not find evidence that switching to e-cigarettes prevented relapse to cigarette smoking. We identified 9.4% of adults categorized as previous-year established smokers had become recent former smokers at follow-up 1. This level was not substantially higher than the 7.4% identified in the 2015 US National Health Interview Survey.^21^ Among these recent former smokers, in both unadjusted and adjusted analyses we found no evidence to support the hypothesis that switching to e-cigarettes reduced relapse to cigarette smoking, whether or not e-cigarettes were used daily. Indeed, the evidence from the PATH study suggests that successful quitting was similar whether the recent former smoker switched to e-cigarettes or to an alternate tobacco product—with relapse being 6% to 9% higher than for those who were tobacco-free. However, switching to receive nicotine from any other tobacco product was associated with a higher rate of requitting for 3 months or more. A further follow-up survey is needed to identify whether this is evidence of a pattern of chronic quitting and relapsing to cigarette smoking or whether it is part of progress toward successful quitting.

Among recent former smokers, the highest prevalence of switching to e-cigarette use was 31% among those who were in the top tertile of tobacco dependence when they were still smoking cigarettes, and the lowest prevalence (approximately 15%) was identified among those who were least dependent and among those in Hispanic or non-White race and ethnicity groups. Those with higher incomes were also more likely to have switched to e-cigarettes. However, there were no differences in switching to e-cigarettes by age, sex, education, or by the duration of the quit attempt prior to follow-up 1.

Randomized trials have focused on e-cigarettes as cessation aids rather than on whether individuals who smoke can switch to e-cigarettes and not relapse to smoking.^22^ Most of these have not reported definitive results.^2,6^ Two recent trials reported that e-cigarettes (with counseling assistance) can help smokers quit: Hajek et al^4^ compared the effectiveness of e-cigarettes vs nicotine replacement therapy and reported that those randomized to e-cigarettes and counseling were more likely to be cigarette abstinent for a year than those randomized to nicotine replacement therapy; Eisenberg et al^5^ reported that volunteers randomized to e-cigarettes and counseling were more likely to be cigarette abstinent at 3 months than a counseling control group. Analyses of the observational PATH study^23^ and similar cohort studies^24^ have also noted that using e-cigarettes to aid quitting is associated with short-term cigarette abstinence, thus corroborating the results of these randomized trials, but that long-term successful quitting has not been demonstrated.

Our study did not focus on a quit attempt, rather it identified recent former smokers who had already switched to an alternative nicotine delivery system and explored whether they could remain cigarette free for at least another year, which is a goal if e-cigarettes are to be a harm reduction alternative. Our sample self-selected their alternate nicotine delivery system, so we adjusted for 19 preidentified potential confounders associated with e-cigarette use.

The PATH study is a large population cohort of a representative sample of the US population with a rigorous methodology.^13^ Our inclusion of a baseline survey to include respondents who currently were smoking in the study sample allowed important potential confounders, such as tobacco dependence, to be measured when the individual was dependent rather than relying on the individual’s recall. Also, each PATH study survey obtained detailed current use of a comprehensive set of tobacco products, allowing our study to evaluate switching to a broad range of alternative tobacco products. While the PATH study did collect biomarkers of tobacco use, these have been analyzed only on select subsamples. In both PATH study wave 1^25^ and wave 2,^26^ biomarker concentrations were in line with reported tobacco product use.

### Limitations

This study had several limitations. Like nearly all population studies, this study was observational, and the exposure variable was not under experimental control, thus limiting causal inference. Accordingly, we followed the recommendations of the National Academies of Science, Engineering, and Medicine for best analytic practices for assessing the effect of e-cigarettes and cessation.^2^ While our analytic design adjusted for many potential confounding variables, there are undoubtedly other variables that are unmeasured confounders and limit causal inference.

## Conclusions

This longitudinal follow-up cohort study of a large representative sample of US recent former smokers showed that switching to e-cigarettes (even on a daily basis) was not associated with helping smokers remain abstinent from cigarettes. Indeed, the evidence suggested that switching to alternate tobacco products by recent former smokers may be associated with increased risk of a relapse to cigarette smoking.

## Notes

Supplement. **eAppendix.** Measurement Detail for Preidentified Study Covariates in PATH Study (With Variable Names) **eTable.** Full Sample Characteristics of PATH Study US Recent Former Cigarette Smokers, According to Use of Non-Cigarette Tobacco Products at Follow-up 1
