# Supplementary material for: Incidence of Cigarette Smoking Relapse Among Individuals Who Switched to e-Cigarettes or Other Tobacco Products
Source: JAMA Netw Open. 2021 Oct 19;4(10):e2128810. doi: 10.1001/jamanetworkopen.2021.28810 (PMC8527352; doi:10.1001/jamanetworkopen.2021.28810)
Supplement: Supplement. — eAppendix. Measurement Detail for Preidentified Study Covariates in PATH Study (With Variable Names) eTable. Full Sample Characteristics of PATH Study US Recent Former Cigarette Smokers, According to Use of Non-Cigarette Tobacco Products at Follow-up 1 [file jamanetwopen-e2128810-s001.pdf]

Supplemental Online Content

Pierce JP, Chen R, Kealey S, et al. Incidence of Cigarette Smoking Relapse Among Quitters Who Switched to e-Cigarettes or Other Tobacco Products. *JAMA Netw Open.* 2021;4(10):e2128810. doi:10.1001/jamanetworkopen.2021.28810

eAppendix. Measurement Detail for Preidentified Study Covariates in PATH Study (With Variable Names)

eTable. Full Sample Characteristics of PATH Study US Recent Former Cigarette Smokers, According to Use of Non-Cigarette Tobacco Products at Follow-up 1

This supplemental material has been provided by the authors to give readers additional information about their work.

eAppendix. Measurement Detail for Preidentified Study Covariates in PATH Study (With Variable Names)

Socio-demographics: Use standard derived variables for age, sex, ethnicity, race, education, and income (First cohort: R01R_A_AGE, R01R_A_SEX, R01R_A_HISP, R01R_A_RACE, R01R_A_EDUC, R01_AM0030; Second cohort: R02R_A_AGE, R02R_A_SEX, R02R_A_HISP, R02R_A_RACE, R01R_A_EDUC, R02_AM0030) Note the variable of education in the second cohort still comes from PATH Wave 1 database, it's not available in Wave 2 database based on our knowledge.

Tobacco dependence index: (First cohort: R01_AN0025, R01_AN0030, R01_AN0065, R01_AN0035, R01_AN0045, R01_AN0085, R01_AN0090, R01_AN0060, R01_AN0095, R01_AN0100, R01_AN0055, R01_AN0050, R01_AN0070, R01_AN0075, R01_AN0080; Second cohort: R02_AN0025, R02_AN0030, R02_AN0065, R02_AN0035, R02_AN0045, R02_AN0085, R02_AN0090, R02_AN0060, R02_AN0095, R02_AN0100, R02_AN0055, R02_AN0050, R02_AN0070, R02_AN0075, R02_AN0080). Variables are combined to derive variables tobacco dependence index for cohort 1 and cohort 2 respectively by calculating the mean of the non-missing scores. Tobacco dependence items take the form of a series of statements on emotional and physical responses to tobacco products (e.g. "I frequently crave {product}", "I usually want to {use product} right after I wake up", "I [would] feel alone without my {product}"). Respondents are asked to rate their level of agreement with each statement on a 5-point scale, where 1="Not true of me at all" and 5="Extremely true of me". Respondents can also answer "don't know" or refuse to answer the question; these are treated as missing responses. Responses are rescaled to a 3-point scale, where 1 (not at all) = 0, 2 or 3 = 50 and 4 or 5 =100, summed and divided by the number of non-missing values.

Cigarette consumption at baseline: Average number of cigarettes now smoked each day (First cohort: R01_AC1021UN, R01_AC1021NN; Second cohort: R02_AC1021UN, R02_AC1021NN). Responses could be reported as cigarettes or packs. For respondents with missingness in those variables, we replaced their cigarette consumption by multiplying average number of cigarettes smoked per day among non-current 30-day smokers (First cohort: R01_AC1023UN, R01_AC1023NN; Second cohort: R02_AC1023UN, R02_AC1023NN) with the number of days smoked in the past 30 days (First cohort: R01_AC1022; Second cohort: R02_AC1022), and divided by 30 days.

Duration of cigarette abstinence: Questions about "How long since you completely quit smoking cigarettes" were identified to derive time of quitting cigarettes (First cohort: R01_AC1009UN, R01_AC1009NN; Second cohort: R02_AC1009UN, R02_AC1009NN).

Insurance status (at Wave 1 or Wave 2): (First cohort: R01_AM0026_01 to R01_AM0026_08; Second cohort: R02_AM0026_01 to R02_AM0026_08): Respondents who reported currently being covered by at least one type of health insurance, including insurance purchased directly or through an employer or union, Medicare, Medicaid, VA, TRICARE or other military health care and Indian Health Insurance, were scored as having insurance coverage. Missing data on all of these variables were coded to "did not have insurance".

External mental health symptoms: Respondents were asked the last time they had experienced any of 7 externalizing (e.g., had a hard time paying attention or listening to instructions at school, work or home, bullied or started physical fights). The number of reports of experiencing such symptoms in the past month or the past 2-12 months was summed and coded into a 3-level severity indicator, with those reporting 0 or 1 symptom scored as Low, 2-3 symptoms scored as Moderate and 4 or more scored as High.

Internal mental health symptoms: Respondents were asked the last time they experienced any of 4 internalizing disorder symptoms: feeling very trapped, lonely, sad, blue, depressed, or hopeless about the future, feeling very anxious, nervous, tense, scared, panicked, or like something bad was going to happen, had sleep problems. The number of reports of experiencing such symptoms in the past month or the past 2-12 months was summed and coded into a 3-level severity indicator, with those reporting 0 or 1 symptom scored as Low, 2-3 symptoms scored as Moderate and 4 or more scored as High.

© 2021 Pierce JP et al. *JAMA Network Open.*

Existence of smoking-related disease: Respondents were asked if they had ever been told by a doctor or health professional that they had any of the listed diseases.
Group A: Heart Disease: High blood pressure (R01_AX0111_01 and R02_AX0111_NB_01), High cholesterol (R01_AX0111_02 and R02_AX0111_NB_02) Congestive heart failure (R01_AX0111_03 and R02_AX0111_NB_03); a stroke (R01_AX0111_04 and R02_AX0111_NB_04); A heart attack (R01_AX0111_05 and R02_AX0111_NB_05); Some other heart condition (R01_AX0111_06 and R02_AX0111_NB_06)
Group B: Respiratory Disease: COPD (R01_AX0119_01 and R02_AX0119_NB_01); chronic bronchitis (R01_AX0119_02 and R02_AX0119_NB_02); emphysema (R01_AX0119_03 and R02_AX0119_NB_03); asthma (R01_AX0119_04 and R02_AX0119_NB_04); some other lung or respiratory condition (R01_AX0119_05 and R02_AX0119_NB_05)
Group C: Cancer: (R01_AX0144 and R02_AX0144 _NB)
"R01_X" variables were used for the first cohort; "R01_X" and "R02_X" variables were combined used for the second cohort;
Smoke-free home: Variables (First cohort: R02_AR1045; Second cohort: R03_AR1045) were used to identify the variable smoke-free home when participants became recent former cigarette smokers. Statement that best describes rules about smoking a combustible tobacco product inside home. It's a 3-point scale from 1 (not allowed anywhere or anytime at all) to 3 (allowed anywhere or anytime at all).
Perceived harmfulness of cigarettes: Respondents were asked "How harmful do you think cigarettes are to health?" and could reply on a 5-point scale from 1 (not at all harmful) to 5 (extremely harmful) (First cohort: R01_AC9050; Second cohort: R02_AC9050).
Relative perceived harmfulness of e-cigarettes: Respondents were asked "Is using e-cigarettes less harmful, about the same, or more harmful than smoking cigarettes?" and could reply on a 3-point scale, where 1=Less harmful, 2=About the same and 3=More harmful (First cohort: R01_AE1099; Second cohort: R02_AE1099).
Exposure to smokers: "In the past 7 days, number of hours that you were in close contact with others when they were smoking." (First cohort: R01_AX0068: Second cohort: R02_AX0068).
Age began regular smoking: (First cohort: R01_AC1007; Second cohort: R01_AC1007 and R02_AC1007_NB).
Cigarette pack-years: (R02R_A_PACKYEARS_CIGS): Calculated by multiplying the number of pack smoked per day by the number of years the respondent smoked regularly, missing values in the number of pack smoked per day were completed by cigarette consumptions calculated above.

© 2021 Pierce JP et al. *JAMA Network Open.*

eTable. Full Sample Characteristics of PATH Study US Recent Former Cigarette Smokers[a], According to Use of Non-Cigarette Tobacco Products[b] at Follow-up 1

Wtd % is the population in row category (for example, among those aged 18-34, 53.5% didn't use tobacco at the exposure assessment, 25.5% used e-cigarette and 46.5% used non-cigarette tobacco product etc.). It extends to all covariates used in the analysis based on those in Tables 1 of the main paper.

			Non-cigarette Tobacco Product Use by Recent Former Smokers					
Variable	Category	Sample size	No tobacco use (n=769)		Any non-cigarette tobacco use (n=459)		Any e-cigarette use[d] (n=286)	
			Wtd %	95% CL	Wtd %	95% CL	Wtd %	95% CL
Age	18-34	546	53.3	47.3, 59.4	46.7	40.6, 52.7	25.8	20.9, 30.6
	35-50	351	66.7	61.3, 72.2	33.3	27.8, 38.7	23.1	17.9, 28.4
	50+	331	72.4	67.6, 77.1	27.7	22.9, 32.4	18.5	13.5, 23.6
Sex	Male	641	56.3	51.7, 60.9	43.7	39.1, 48.3	25.1	21.1, 29.0
	Female	587	71.6	67.5, 75.8	28.4	24.2, 32.5	19.9	15.7, 24.1
Education	Less than high school	254	66.6	60.5, 72.8	33.4	27.2, 39.5	19.1	13.9, 24.3
	High school graduate	258	62.4	54.9, 69.8	37.6	30.2, 45.1	20.4	14.8, 26.0
	Some college or higher	707	61.8	57.4, 66.2	38.2	33.8, 42.6	25.0	20.9, 29.1
	Missing	9	69.6	*	30.4	*	30.4	*
Ethnicity	Hispanic	173	77.2	70.5, 83.9	22.8	16.1, 29.5	15.0	9.2, 20.9
	Non-Hispanic	1042	60.7	57.0, 64.4	39.3	35.6, 43.0	24.1	20.6, 27.5
	Missing	13	85.5	*	14.5	*	8.0	*
Race	White	935	61.5	57.4, 65.7	38.5	34.3, 42.6	25.0	21.2, 28.8
	Others	264	66.8	60.7, 72.9	33.2	27.1, 39.3	14.9	9.5, 20.2
	Missing	29	83.9	73.2, 94.7	16.1	5.3, 26.8	10.6	1.1, 20.1
Income (US$)	< 35000	625	65.7	61.8, 69.6	34.3	30.4, 38.2	19.3	16.3, 22.3
	>= 35000	530	58.9	53.7, 64.1	41.1	35.9, 46.3	27.5	22.5, 32.4
	Missing	73	71.9	59.9, 84.0	28.1	16.0, 40.1	14.7	4.3, 25.0
Tobacco dependence index[c]	0-33.3	441	71.3	66.3, 76.3	28.7	23.7, 33.7	14.9	10.9, 18.9
	33.4-66.7	490	61.0	55.3, 66.6	39.0	33.4, 44.7	25.4	20.3, 30.5

© 2021 Pierce JP et al. *JAMA Network Open.*

	66.8-100	295	52.6	46.0, 59.2	47.4	40.8, 54.0	31.3	25.0, 37.7
	Missing	2	100.0	*	0.0	*	0.0	*
Cigarette consumption at baseline (pack)	< 1	939	63.0	59.5, 66.6	37.0	33.4, 40.5	22.3	19.1, 25.5
	>= 1	235	59.8	52.6, 67.1	40.2	32.9, 47.4	29.3	22.8, 35.8
	Missing	54	74.0	61.1, 86.8	26.0	13.2, 38.9	6.3	0.3, 12.3
Duration of cigarette abstinence (days)	<90	442	60.8	55.5, 66.0	39.3	34.0, 44.5	24.3	19.4, 29.1
	>=90	775	64.4	60.4, 68.4	35.6	31.6, 39.6	22.1	18.3, 25.8
	Missing	11	40.9	*	59.1	*	21.6	*
Insurance Status	Yes	975	63.1	59.3, 66.9	36.9	33.1, 40.7	22.9	19.3, 26.5
	No	245	63.1	56.6, 69.6	36.9	30.4, 43.4	21.8	16.5, 27.1
	Missing	8	37.6	*	62.4	*	38.7	*
External mental health symptoms	Low	725	64.8	60.8, 68.8	35.2	31.2, 39.2	21.0	17.2, 24.8
	Moderate	320	59.8	52.0, 67.5	40.2	32.5, 48.0	28.3	22.1, 34.5
	High	183	60.3	51.9, 68.7	39.7	31.3, 48.1	21.0	14.6, 27.5
Internal mental health symptoms	Low	674	66.6	62.6, 70.5	33.4	29.5, 37.4	19.6	15.9, 23.4
	Moderate	288	59.6	52.1, 67.1	40.4	32.9, 47.9	24.1	18.4, 29.9
	High	266	56.2	48.6, 63.8	43.8	36.2, 51.4	30.4	23.6, 37.2
Existence of smoking-related disease	Yes	590	66.5	62.6, 70.5	33.5	29.5, 37.4	22.1	17.9, 26.2
	No	638	59.6	54.7, 64.4	40.4	35.6, 45.3	23.5	19.7, 27.4
Smoke-free home	Yes	922	65.6	62.0, 69.2	34.4	30.8, 38.0	22.6	19.4, 25.9
	No	300	55.4	49.0, 61.7	44.6	38.3, 51.0	23.4	17.2, 29.6
	Missing	6	31.1	*	68.9	*	25.3	*
Perceived harmfulness of cigarettes	Not to somewhat harmful	255	63.5	57.9, 69.1	36.5	30.9, 42.1	21.6	15.1, 28.1
	Very/extremely harmful	972	62.8	58.8, 66.8	37.2	33.2, 41.2	23.1	19.6, 26.6
	Missing	1	0.0	*	100.0	*	100.0	*
Relative perceived harmfulness of e-cigarettes	1 = Less harmful	612	54.4	50.2, 58.7	45.6	41.3, 49.8	32.5	28.1, 36.9
	2 = About the same	470	67.9	63.1, 72.7	32.1	27.3, 36.9	15.5	11.6, 19.3
	3 = More harmful	84	76.7	65.5, 87.8	23.3	12.2, 34.5	8.5	2.4, 14.6
	Missing	62	84.7	74.9, 94.5	15.3	5.5, 25.1	7.8	0.3, 15.4
Exposure to smokers (in the past 7 days)	<=10 hours	896	64.7	61.3, 68.2	35.3	31.8, 38.7	21.7	18.4, 25.0
	>10 hours	315	58.1	51.5, 64.7	41.9	35.3, 48.5	25.7	20.1, 31.2
	Missing	17	52.4	*	47.6	*	31.5	*
	18+	590	67.7	63.1, 72.3	32.3	27.7, 36.9	20.7	16.4, 25.0

© 2021 Pierce JP et al. *JAMA Network Open.*

Age began regular smoking	< 18	599	56.2	51.2, 61.2	43.8	38.8, 48.8	26.4	22.4, 30.5
	Missing	39	80.2	66.9, 93.5	19.8	6.5, 33.1	8.3	0.0, 17.8
Cigarette pack-years	<= 20	849	59.9	55.8, 63.9	40.1	36.1, 44.2	24.0	20.5, 27.5
	21-35	136	64.1	54.7, 73.6	35.9	26.4, 45.3	27.4	18.0, 36.8
	> 35	162	66.5	59.3, 73.7	33.5	26.3, 40.7	22.3	15.3, 29.4
	Missing	81	80.2	71.5, 88.8	19.8	11.2, 28.5	7.2	1.1, 13.3

Abbreviations: PATH, Population Assessment of Tobacco and Health; Wtd, Weighted US population estimate; CL, Confidence Limit.

[*] Confidence limits of those categories with sample size less than 20 were not estimated.

[a] Recent Former Smoker: those who were not smoking cigarettes at Follow-up 1 but who were Established Smokers at Baseline.
[b] Non-cigarette tobacco use include any use of e-cigarette, other e-products, cigar, cigarillo, filtered cigar, pipe, hookah, snus or smokeless tobacco.
[c] Tobacco Dependence Index tertiles based on Strong et al (ref #9).
[d] E-cigarette use is a subgroup of "any non-cigarette tobacco use"

© 2021 Pierce JP et al. *JAMA Network Open.*
